# Does the application of autologous injectable Platelet-Rich Fibrin (i-PRF) affect the patient’s daily performance during the retraction of upper canines? A single-centre randomized split-mouth controlled trial

**DOI:** 10.1186/s12903-023-03646-z

**Published:** 2023-11-17

**Authors:** Talar Zeitounlouian, Rania Haddad, Bassel Brad, Muhammed Al-Huda Ballouk, Piotr Fudalej

**Affiliations:** 1 Department of Orthodontics, Faculty of Dentistry, Damascus University, Damascus, Syria; 2 Department of Oral and Maxillofacial Surgery, Faculty of Dentistry, Damascus University, Damascus, Syria; 3 Department of Paediatric Dentistry, Faculty of Dentistry, Damascus University, Damascus, Syria; 4 Department of Orthodontics, Institute of Dentistry, Medical Faculty, Jagiellonian University, Kraków, Poland; 5 Department of Orthodontics, Institute of Dentistry and Oral Sciences, Faculty of Medicine and Dentistry, Palacký University Olomouc, Olomouc, Czech Republic; 6 Department of Orthodontics, School of Dental Medicine, Medical Faculty, University of Bern, Bern, Switzerland

**Keywords:** Injectable Platelet-Rich Fibrin (i-PRF), Maxillary canine retraction, Patient-Reported Outcome Measures (PROMs), Quality of Life (QoL), Visual Analogue Scale (VAS)

## Abstract

**Background:**

Previous studies have assessed different aspects concerning the applications of i-PRF in the oral cavity. However, nothing is known regarding patients’ perceptions of the injection of autologous platelet-rich fibrin (i-PRF).

**Objectives:**

To investigate patients’ perceptions after injecting platelet-rich fibrin (i-PRF) in the course of retracting upper canines.

**Methods:**

Twenty-one patients, whose treatments required extractions of both upper first premolars, were recruited. Extraction side was randomly allocated to the intervention or control sides. After the alignment phase, i-PRF was injected twice with a one-month interval on the buccal and palatal aspects of the extraction sites (intervention side). Patients’ perceptions were evaluated with two questionnaires: the first was used to assess the level of pain, discomfort, swelling, eating and swallowing difficulties as well as jaw movement restriction after 1 h (T1), 2 h (T2), 6 h (T3), 24 h (T4) and 48 h (T5) of the second injection; the second questionnaire was used to assess the acceptance of the i-PRF injection and overall satisfaction with this technique at the end of canine retraction phase. Visual Analogue Scale (VAS) was adopted for this purpose. Wilcoxon Signed Rank Test was used to compare between both sides at all time points while Friedman’s Test was the selected test for detecting variables’ changes over time. Post-hoc Wilcoxon Matched-Pairs Signed-Rank Tests were applied when any of the results were significant. As to the multiplicity of tests, Bonferroni Correction was implemented.

**Results:**

Pain and swelling levels were significantly higher on the experimental compared to the control sides at T1, T2, and T3 (*P* < 0.05), whereas they declined sharply and went back to almost normal values at T4 (after 24 h). At T5 they were 0. Discomfort and difficulty in mastication on intervention sides were significant only at T1 and T2. Pain, swelling, and chewing difficulties were significant (*P* < 0.001) during the 4 assessed time points. The increase was insignificant regarding swallowing difficulties and jaw movement limitations at all time intervals.

**Conclusions:**

Injecting autologous (i-PRF) during orthodontic canine retraction is a well-perceived and well-tolerated method due to the limited discomfort which significantly diminishes 24 h afterwards.

**Trial’s registration:**

ClinicalTrials.gov (Identifier Number: NCT03399422. 16/01/2018).

## Introduction

The true efficacy of any treatments under question is something of great importance, definitely. However, it is not only the efficacy of the intervention but rather also the resulting Quality of Life (QoL) that matters. The higher related QoL levels mean that the procedure will meet the patients’ acceptance and the healing will be promoted. Patient-Reported Outcome Measures (PROMs) are real authentic manifestations of the QoL concept, representing the resulting interaction between the patients and the techniques or materials used.

Orthodontic treatment of adult patients is frequently challenging because of their higher expectations regarding aesthetics and comfort. Moreover, fast completion of the orthodontic treatment is one of priorities of adults [1, 2] especially in situations when tooth extraction is necessary [3]. This requirement is difficult to meet due to the decreased bone turnover and increased bone maturity in adults [4]. Therefore, methods of acceleration of the Orthodontic Tooth Movement (OTM) have become the subject of much research in the last decades. Most of them target the process of remodelling of the alveolar bone and periodontal ligament (PDL). They aim, also, to avoid the adverse effects resulting from long and extended orthodontic treatment durations, such as root resorption, white spot lesions, caries, and periodontal problems [4–7] These methods are divided into surgical (osteotomy, corticotomy, corticision, piezocision, micro-osteo-perforation, dentoalveolar distraction osteogenesis, periodontal distraction and surgery first) and non-surgical (self-ligating brackets, medications, photo-biomodulation, electromagnetic field, electrical currents and vibration) [8–10] with the former regarded as more effective [11]. However, they are invasive in nature and are usually associated with pain, oedema, and occasional loss in periodontal support of the tooth. All of the aforementioned can be critical deterrents to the orthodontic treatment and are in fact common causes for treatment discontinuation [8]. Consequently, biomaterials such as platelet-rich plasma (PRP) and platelet-rich fibrin (PRF) have been recently introduced as better alternatives to the surgical interventions and their efficacy has been tested in accelerating orthodontic tooth movement in previous studies trying to overcome the invasive surgical hazards [12–18]. Their potential is mainly attributed to the high contents of growth factors they have which play crucial roles in wound healing and bone regeneration [19, 20]. These biomaterials have been widely used in both dental and medical fields because of their therapeutic effects [21, 22]. Unfortunately, researchers have studied the aforenamed alternative techniques without paying enough attention to their detrimental effects, nor pain levels that could threaten patients’ cooperation in terms of attending their appointments, taking care of their appliances, following the clinician’s instructions [23] and can finally lead them to refuse or cease the orthodontic treatment [24, 25].

Although injectable platelet-rich fibrin (i-PRF) is considered to be a promising biomaterial [21], the scientific evidence is still lacking in terms of patient-reported outcome measures (PROMs) in the orthodontic field according to the systematic review [26] that pointed out only a single study [14] which recorded pain scores combined when using the PRP injection. Moreover, a later systematic review and meta-analysis [27] revealed a huge lack in the studies dealing with the pain and discomfort associated with the use of condensed platelets (platelet-rich concentrates) and there are only 3 articles that discussed the accompanied pain and it was addressed on its own (without any other variables) [14, 28, 29]. On top of that, these 3 papers were based on the use of PRP (not i-PRF). No previous studies have investigated the levels of pain, discomfort, swelling, chewing difficulties, swallowing difficulties, jaw movement limitation, satisfaction, which experience is harder, patients’ recommendations, all, and not only “just pain”. In other words, the PROMs associated with the application of the injectable platelet-rich fibrin (i-PRF) while retracting upper canines in class II division I patients as the main aim of the study. Accordingly, having insignificant differences between both sides regarding the measured variables referred to the null hypothesis.

## Materials and methods

### Study design and sample

**Fig. 1:**
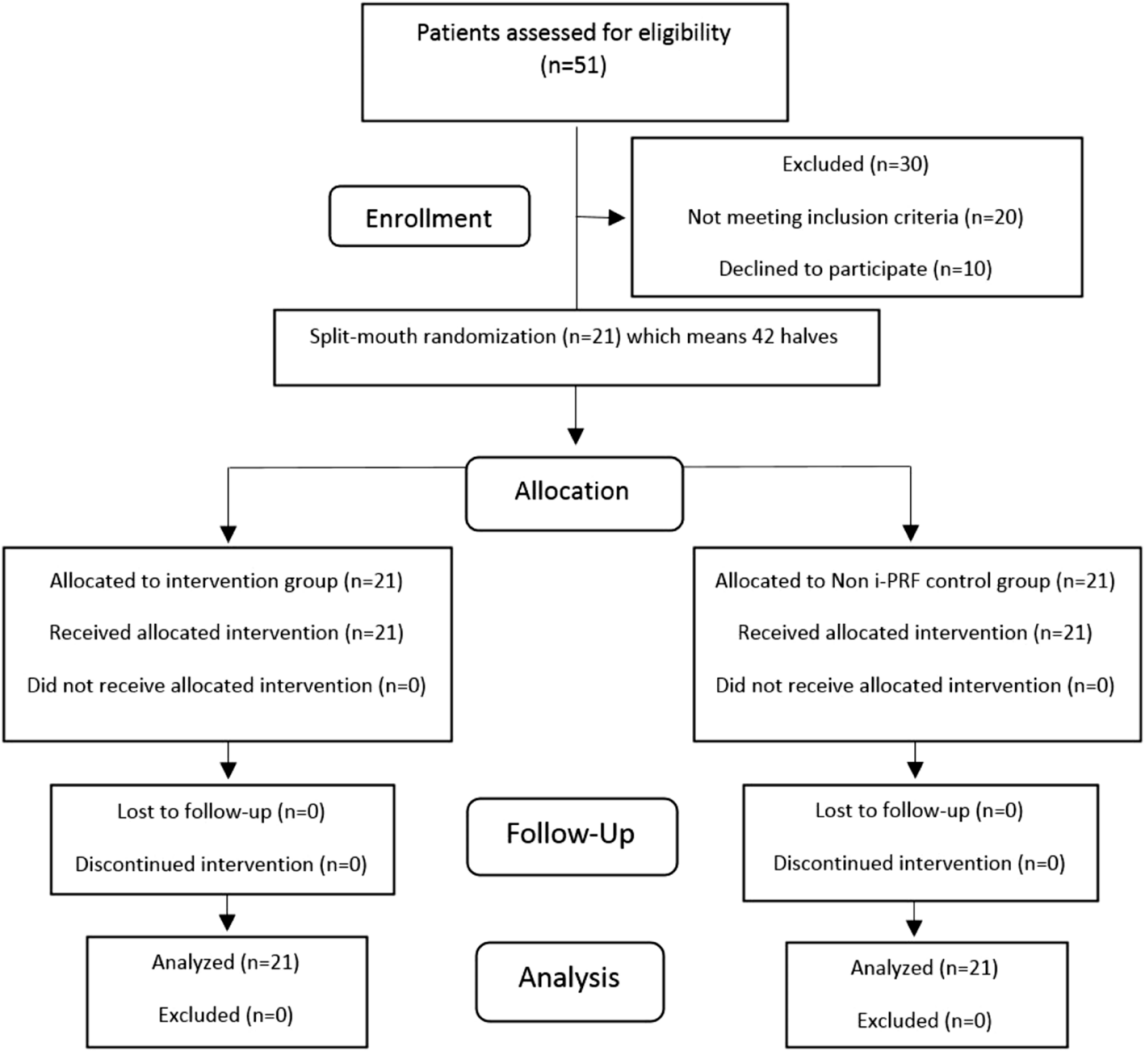
The CONSORT flow chart

It was a randomized split mouth clinical trial with 1:1 allocation ratio to intervention and control sides conducted in the Department of Orthodontics at the Faculty of Dentistry, Damascus University. The study was approved by the institutional review board (IRB) and ethical review committee of Damascus University (N. 2473). The CONSORT (Consolidated Standards of Reporting Trials) statement was followed as a guide for this study which was registered at Clinicaltrials.gov with the identifier number (NCT03399422). The recruited study participants were patients presenting to the Department of Orthodontics, Faculty of Dentistry, Damascus University. Patients’ inclusion and follow-up is shown in the CONSORT flow chart (Fig. 1).

The sample size was calculated to investigate the significant differences in pain perception between both sides based on a split-mouth design previous study [28] and with 90% of study power and 5% of permissible α error using G*Power 3.1.3 software (Heinrich-Heine-Universitӓt, Düsseldorf, Germany). Therefore, 21 participants were recruited in this study. The total duration of the study was 10 months.

### Inclusion and exclusion criteria

*Inclusion criteria were*: patients aged 16–28 years with class II division I malocclusions and mild to moderate skeletal discrepancies (ANB ≤ 7) requiring bilateral maxillary first premolars extractions; crowding ≤ 3; OJ < 10; no tooth loss except third molars; normal to vertical growth pattern; no transverse discrepancy; no systemic diseases; good oral hygiene (Gingival Index < 1, Plaque Index < 1, both according to Silness and Löe) [30], and normal platelet count.

*Exclusion criteria were*: patients taking anticoagulants or medications that interfere with orthodontic tooth movement (NSAIDS, Bisphosphonates, and Corticosteroid), smokers, bony defects observed radiographically, previous history of orthodontic treatment.

The purpose and methods of the study were comprehensively clarified to the potential participants who met the inclusion criteria. After ensuring the patients’ compliance and acceptance, the patients and/or their legal guardians for those who were under 18 years old, were asked to sign an informed consent.

### Randomization and blinding

Computer-generated random numbers were used for randomization of the right and left extraction sides to either the experimental side (i-PRF) and control side (non i-PRF), with a 1:1 allocation ratio. The randomization was done by a research assistant who was not involved in this trial. Blinding was only applicable in the data analyses phase.

### The intervention

**Fig. 2:**
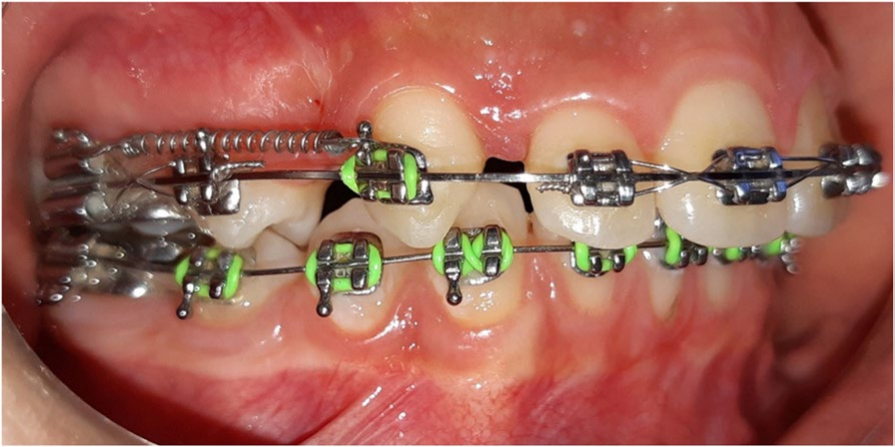
Canine retraction using NiTi closed coil spring

**Fig. 3:**
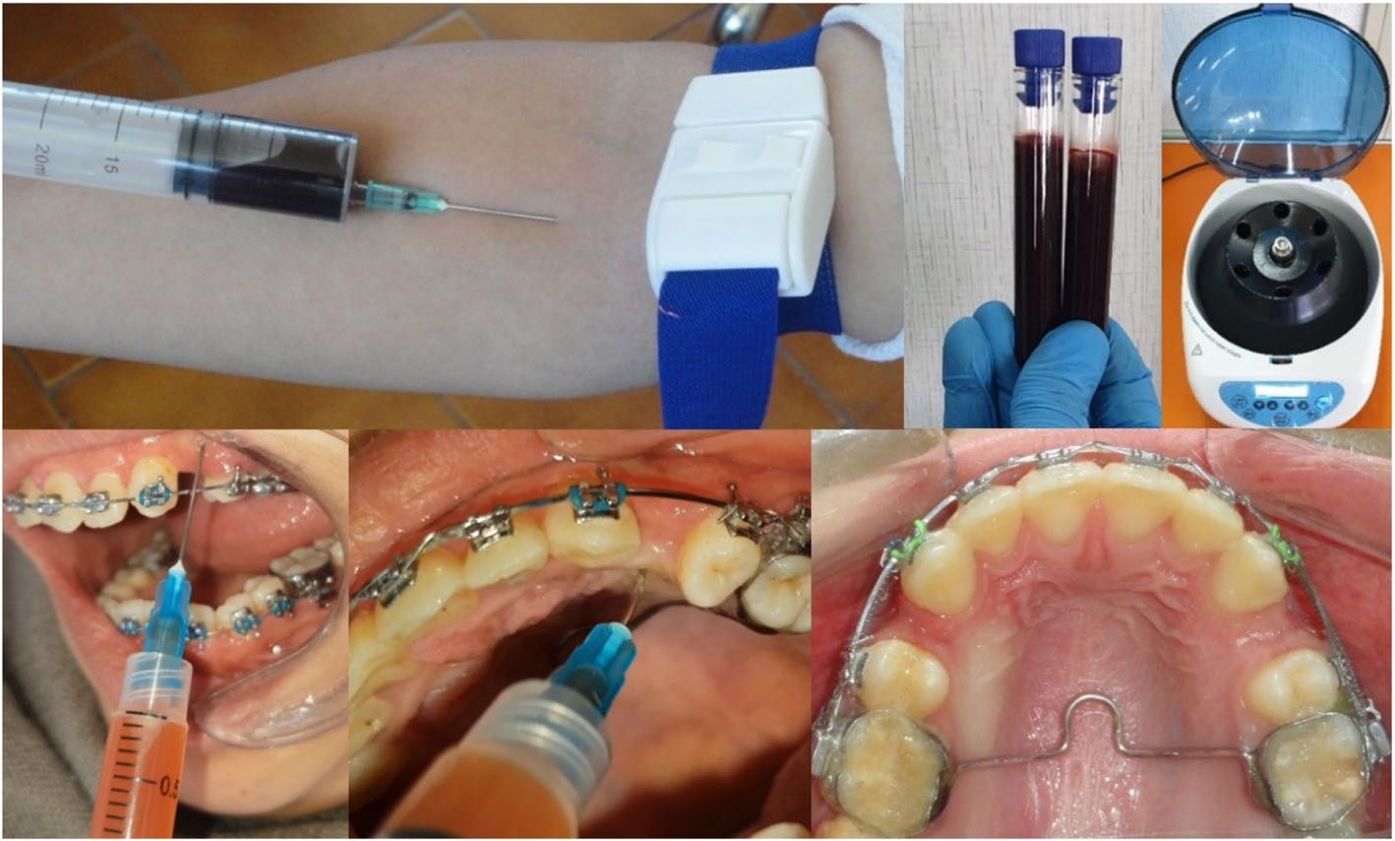
The preparation along with the application and post-application of the i-PRF

The intervention was an injection of i-PRF on one of the extraction sides in a predetermined moment of the standardized orthodontic treatment, which comprised; fixed orthodontic appliances with MBT.022 inch slot (Votion, Ortho Technology, West Columbia, SC, USA), initial archwire sequence was: 0.014-in NiTi (or 0.016-in NiTi depending on the amount of crowding), 0.016*0.022-in NiTi, and, 0.017*0.025-in NiTi; extraction of the maxillary first premolars just before the insertion of 0.019*0.025-in SS archwire; canine retraction was achieved with closed nickel-titanium coil springs with 150 g of force per side (Fig. 2); 20 mL blood were drawn from each patient and centrifuged (700 rpm within 3 min) [31] by using (HW6C, HWLAB® Mini Combo Centrifuge, ZheJiang, China) and dry sterile glass tubes without any additives to get approximately 3 mL of the yellow orange upper portion (the i-PRF). The i-PRF was injected twice: at the moment of initiating the canine retraction and 1 month later, both at the area of extracted upper first premolar of the intervention side after topical anesthetization with 8% lidocaine spray. Two mL were injected on the buccal and 1 mL on the palatal intervention sides the same way as in the method used for local infiltrative anaesthesia (Fig. 3). No medications were prescribed following the injection. All clinical procedures—orthodontic treatments and injections—were done by the same investigator (TZ).

### Questionnaires

**Fig. 4:**
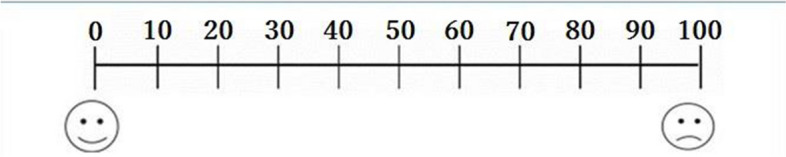
Visual Analogue Scale (VAS) used in this study to evaluate patient-reported outcome measures other than personal satisfaction

Two questionnaires (Q1 and Q2) were administered following a comprehensive explanation of the purpose of the survey: the Q1 aimed to record the levels of pain, discomfort, swelling, difficulties in mastication, difficulties in swallowing and jaw movement restriction using a 100-mm Visual Analogue Scale (VAS) – Fig. 4, where 0 mm denoted the most favourable situation (e.g. no pain) and 100 mm denoted the least favourable situation (e.g. the worst pain ever). All participants were asked to fill in the Q1 at 5 time points: 1 h (T1), 2 h (T2), 6 h (T3), 24 h (T4) and 48 h (T5) after the 2^nd^ i-PRF injection.

**Fig. 5:**
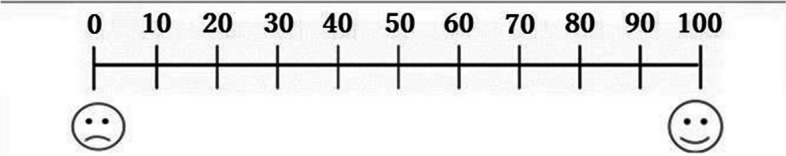
Visual Analogue Scale (VAS) used in this study to evaluate patients’ personal satisfaction

The Q2 questionnaire consisted of 3 questions aiming to assess patient’s satisfaction with the procedure and a probability to recommend this procedure to patient’s family and/or friends. The Q2 was administered at the end of canine retraction phase (Table 1). A 100 mm VAS was used, where 0 mm denoted the least satisfaction (e.g. not happy with the experience at all) and 100 mm denoted complete satisfaction (e.g. totally happy with it)—Fig. 5.

**Table 1:** The list of questions asked to the patients in both questionnaires

	**N**^**o**^	**Question**^**a**^
Questionnaire 1	1	How much pain do you feel at the injection side?
	2	How much pain do you feel at the control side?
	3	Do you experience discomfort at the injection side?
	4	Do you experience discomfort at the control side?
	5	Do you experience swelling at the injection side?
	6	Do you experience swelling at the control side?
	7	Do you experience chewing difficulties at the injection side?
	8	Do you experience chewing difficulties at the control side?
	9	Do you experience swallowing difficulties?
	10	Do you experience jaw movement limitations?
Questionnaire 2	11	Are you satisfied with this procedure?
	12	What was more disturbing to you-the extraction of the premolars or the injection? The extraction—The injection – Both
	13	Would you advise this procedure to a friend? Yes/No

^a^The first ten questions were included in the first questionnaire while the remaining ones in the second questionnaire

### Statistical analysis

Statistical analysis was accomplished using the IBM SPSS version 25 (SPSS Inc., Chicago, III, USA), probability values equal or less than 0.05 were considered significant. The analysis was performed by one of the researchers who was blinded to study results. Non-parametric tests were used to analyse the data that were not normally distributed and in particular Wilcoxon Signed Rank Test to compare the levels of pain, discomfort, swelling and chewing difficulties between the two sides. Friedman’s Test was the selected test for detecting variables’ changes over time. The Post-hoc Wilcoxon Matched-Pairs Signed-Rank Tests were applied when any of the results were significant. Bonferroni Correction was adopted for the purpose of multiplicity of tests.

## Results

Twenty-one patients aged 16–28 years (mean age 20.9, SD =  ± 3.9 years) participated in this study. There were no changes to the study protocol after trial commencement. The main outcome measure—the time of canine retraction—was described in our previous study. In summary, i-PRF injection failed to reduce the duration of canine retraction because it significantly accelerated upper canine retraction only during the 2^nd^ month of the retraction period (an acceleration rate 31.7%), while there were no differences in the rate of canine movement on intervention and control sides in the remaining months of retraction phase [17].

Descriptive statistics of responses to the Q1 questionnaire are shown in (Tables 2 and 3). Please note that, since all values taken at T5 (48 h after injection) were 0, they were not tabulated.

**Table 2:** Descriptive statistics study of the levels of pain, discomfort, swelling, difficulty in chewing on the experimental and control sides as well as the *P*-Values for significance tests

**Time**	**Variable**	**Experimental Side (*n* = 21)**					**Control Side (*n* = 21)**					*P*-Value^†^
		**Mean**	**SD**	**Min**	**Max**	**Median (IQR)**	**Mean**	**SD**	**Min**	**Max**	**Median (IQR)**	
**T1**	**Pain**	24.28	17.48	0.00	60.00	20.00 (15.00–40.00)	3.80	8.64	0.00	30.00	0.00 (0.00–0.00)	< 0.001***
	**Discomfort**	23.80	17.45	0.00	60.00	20.00 (10.00–35.00)	6.19	10.23	0.00	40.00	0.00 (0.00–10.00)	< 0.001***
	**Swelling**	25.23	16.00	0.00	60.00	20.00 (15.00–30.00)	2.38	6.24	0.00	20.00	0.00 (0.00–0.00)	< 0.001***
	**Difficulty in Chewing**	14.76	18.87	0.00	70.00	10.00 (0.00–20.00)	9.04	17.86	0.00	70.00	0.00 (0.00–10.00)	0.016*
**T2**	**Pain**	15.23	15.69	0.00	50.00	10.00 (0.00–25.00)	2.38	7.68	0.00	30.00	0.00 (0.00–0.00)	0.002**
	**Discomfort**	16.19	16.27	0.00	50.00	10.00 (0.00–30.00)	4.04	7.68	0.00	20.00	0.00 (0.00–5.00)	0.003**
	**Swelling**	17.14	15.21	0.00	50.00	20.00 (0.00–30.00)	0.47	2.18	0.00	10.00	0.00 (0.00–0.00)	0.001**
	**Difficulty in Chewing**	16.66	19.83	0.00	70.00	20.00 (0.00–30.00)	8.09	15.03	0.00	60.00	0.00 (0.00–15.00)	0.017*
**T3**	**Pain**	5.23	10.30	0.00	40.00	0.00 (0.00–10.00)	1.42	6.54	0.00	30.00	0.00 (0.00–0.00)	0.023*
	**Discomfort**	8.57	15.25	0.00	60.00	0.00 (0.00–20.00)	3.80	10.23	0.00	40.00	0.00 (0.00–0.00)	**0.112**
	**Swelling**	6.19	9.73	0.00	30.00	0.00 (0.00–15.00)	0.95	4.36	0.00	20.00	0.00 (0.00–0.00)	0.015*
	**Difficulty in Chewing**	9.04	16.70	0.00	70.00	0.00 (0.00–15.00)	8.09	15.69	0.00	60.00	0.00 (0.00–10.00)	**0.577**
**T4**	**Pain**	2.38	8.89	0.00	40.00	0.00 (0.00–0.00)	0.47	2.18	0.00	10.00	0.00 (0.00–0.00)	**0.180**
	**Discomfort**	3.33	6.58	0.00	20.00	0.00 (0.00–5.00)	3.33	9.66	0.00	40.00	0.00 (0.00–0.00)	**1.00**
	**Swelling**	0.00	0.00	0.00	0.00	0.00 (0.00–0.00)	0.00	0.00	0.00	0.00	0.00 (0.00–0.00)	**1.00**
	**Difficulty in Chewing**	2.38	7.00	0.00	30.00	0.00 (0.00–0.00)	2.85	7.83	0.00	30.00	0.00 (0.00–0.00)	**0.317**

T1 is 1 h after the injection; T2 is 2 h after the injection; T3 is 6 h after the injection; T4 is 24 h after the injection

^*^P < 0.05

^**^P < 0.01

^***^P < 0.001

^†^Wilcoxon Signed-Rank Test

**Table 3:** Descriptive statistics study of the difficulty in swallowing and jaw movement limitation reported by the patients as well as Friedman’s Test to show any significance between the four time points

**Variable**	**Time**	**Mean**	**SD**	**Min**	**Max**	**Median (IQR)**	***P*-Value**^**†**^
**Difficulty in Swallowing**	**T1**	1.90	6.01	0.00	20.00	0 (0.00)	**0.112**
	**T2**	0.72	2.43	0.00	10.24	0 (0.00)	
	**T3**	0.00	0.00	0.00	0.00	0 (0.00)	
	**T4**	0.00	0.00	0.00	0.00	0 (0.00)	
**Jaw Movement Limitation**	**T1**	2.38	7.68	0.00	30.00	0 (0.00)	**0.112**
	**T2**	1.45	4.82	0.00	20.00	0 (0.00)	
	**T3**	0.00	0.00	0.00	0.00	0 (0.00)	
	**T4**	0.00	0.00	0.00	0.00	0 (0.00)	

T1 is 1 h after the injection; T2 is 2 h after the injection; T3 is 6 h after the injection; T4 is 24 h after the injection

^†^Friedman’s Test

Table 2 demonstrated that the mean values of pain, discomfort, swelling, and difficulties in chewing were higher on the interventional side when compared to the control at T1, T2, and T3. However, statistically significant differences were only at T1, T2, T3 for pain (*P* < 0.001, *P* = 0.002, *and P* = 0.023, respectively) and swelling levels (*P* < 0.001, *P* = 0.001, and *P* = 0.015, respectively), while discomfort and chewing difficulties were different between sides at T1 and T2 only (*P* < 0.001, *P* = 0.003 for discomfort; *P* = 0.016, *P* = 0.017 for chewing difficulties). At T4 the differences between both sides were not statistically significant (*P* > 0.05). No significant differences were found in terms of age and gender relationships with pain and discomfort after the implementation of Spearman Correlation Coefficient and Kruskall-Wallis Tests respectively (Table 4).

**Table 4:** Significance tests of pain and discomfort in relation to age and gender as well as the *P*-Values for significance tests

**Variable**	**Time**	**Age**	**Gender**
		***P*-Value**^†^	***P*-Value**^††^
**Pain**	**T1**	0.909	0.066
	**T2**	0.766	0.134
	**T3**	0.116	0.534
	**T4**	0.290	0.418
**Discomfort**	**T1**	0.682	0.147
	**T2**	0.089	0.445
	**T3**	0.947	0.459
	**T4**	0.916	0.740

T1 is 1 h after the injection; T2 is 2 h after the injection; T3 is 6 h after the injection; T4 is 24 h after the injection

^†^Spearman Correlation Coefficient Test

^††^Kruskall-Wallis Test

Difficulties in swallowing and jaw movement limitation were comparable at all timepoints (Table 3). It means that injection of i-PRF didn’t affect these parameters at any point in time.

Regarding longitudinal (i.e. within the group) changes of variables between the 4 time-points, the levels of pain, discomfort, swelling, and difficulty in chewing were significantly different between points in time (*P*-value < 0.001) according to Friedman’s Test. Post-hoc pairwise comparisons with Bonferroni’s adjustment of alpha level for values that showed significant differences are presented in Table 5. On the contrary, the difficulties in swallowing as well as jaw movement limitation showed insignificant differences (*P*-value > 0.05) (Table 3).

**Table 5:** Significance tests results for pairwise comparisons between the four evaluated times for patient-reported outcome measures in the injectable platelet-rich fibrin group

**Time**		**Pain**	**Discomfort**	**Swelling**	**Difficulty in chewing**
**T1**	**T1-T2**	0.001*	0.018	0.001*	0.102
	**T1-T3**	< 0.001*	0.002*	< 0.001*	0.028
	**T1-T4**	< 0.001*	0.001*	< 0.001*	0.003*
**T2**	**T2-T3**	0.003*	0.021	0.001*	0.014
	**T2-T4**	0.002*	0.001*	0.001*	0.003*
**T3**	**T3-T4**	0.063	0.042	0.016	0.010

Wilcoxon Signed-Ranks Tests were employed for pairwise comparisons, Bonferroni’s Adjustment of Alpha Level (i.e., 0.05/6 = 0.008)

T1 is 1 h after the injection; T2 is 2 h after the injection; T3 is 6 h after the injection; T4 is 24 h after the injection

^*^Significant difference when *P* < 0.008

Satisfaction levels of the injection were promising and decent enough (75.71 ± 27.85) as shown in Table 6. The percentage of the patients in terms of feeling disturbed from the extraction of upper premolars was higher 80.95% compared to the injection 14.29% while 4.76% admitted that both procedures were equally annoying and unpleasant. Moreover, this technique was recommended to their friends by the majority (85.71%) of participants.

**Table 6:** Descriptive statistics study of the levels of satisfaction with the injection of i-PRF

**Variable**	**Mean**	**SD**	**SE**	**Min**	**Max**	**Median (IQR)**
**Satisfaction level**	75.71	27.85	6.07	20.00	100.00	90.00 (45.00–100.00)

## Discussion

The application of platelet rich fibrin to facilitate orthodontic tooth movement has not been thoroughly investigated regarding its effect on patients’ daily activities. To the best of our knowledge, there were no previous studies that evaluated patients’ perceptions associated with i-PRF injection during orthodontic treatment. Hence, in this study we assessed pain, discomfort, swelling, and difficulty in mastication and swallowing as well as limitation in jaw movement after application of i-PRF during retraction of the upper canines. Moreover, we evaluated patients’ satisfaction with the procedure. We used VAS to measure the variables that are subjective in nature because it is a very reliable and easy method in addition to its wide usage in other previous studies [14, 32, 33].

The low-speed centrifugation protocol (700 rpm within 3 min) was adopted to obtain the i-PRF [31, 34, 35] because it has several advantages such as, higher rates of regenerative cells and growth factors [36]. In addition, it provides more natural and gradual transformation leading to increased cytokines integrity as well as leukocyte proportions in the fibrin network which in turn increases the duration of cytokines secretion and growth factors release and subsequently the i-PRF efficiency when compared to the conventional form of PRF [37–39]. Furthermore, the injectable version of the PRF enabled us to apply it immediately prior to the initiation of canine retraction in an attempt to get the best possible outcomes.

PRF is the second generation of platelet concentrates that has the advantage of gradual release of growth factors that last up to 28 days [19, 40, 41]. As a result, i-PRF was injected twice with a one-month interval unlike other studies in which different administration frequencies were followed, for example: Karakasli et al. and Erdur et al. [34, 35] applied the i-PRF twice with a 2-week interval in maxillary incisor and canine retraction cases over a follow-up period of one and three months respectively, unlike Karsi and Baka’s study in which the injections of i-PRF were repeated after 4 and 8 weeks of the first delivery [42]. On the contrary, Ibrahim et al. [43] injected the i-PRF only once during upper canine distalization, likewise Rokia et al. [44] administered the i-PRF at the beginning of levelling and alignment stage with no repetitions.

Patients’ responses regarding pain, discomfort, swelling, and difficulty in mastication and swallowing as well as limitation in jaw movement after the second injection were used for analysis because we wanted patients to have prior experience with injection. In this way, we wanted to reduce the role of stress during the response, especially immediately after the injection. By analysing the responses after the second injection, we obtained more reliable information about how patients felt. Additionally, none of the participants used any kind of analgesics what ensured the reliability of the answers.

Despite the differences between the experimental and control sides in the perception of injection-related “stress” for most variables and at most assessment timepoints, the mean values of the studied variables were relatively low even 1 h after injection (T1). For example, our data demonstrated that the levels of pain on the experimental side were statistically significantly higher than on the control one at T1, T2, T3 (*P* < 0.05). Nevertheless, “unpleasantness” caused by pain at T1 was judged as relatively mild. In general, increased pain level could be related to the gingival trauma after injection as well as the simultaneous application of orthodontic force – we used coil springs to move canines—that might cause some irritation and discomfort [45]. Moreover, the general fear of using needles and syringes in the treatment process could be an inevitable fact in any society [46]. Quickly decreasing levels of pain, discomfort and other variables could be caused by the anti-inflammatory properties of the substance that has been proven by many studies in which they used the PRF as a palliative material that help reduce the associated post-operative pain during invasive surgical interventions or third molar extractions [47, 48].

Our results agreed with the findings of the study by Liou [49] who assessed the associated pain and discomfort after application of PRP. It is worth mentioning, however, before going on further, that Liou didn’t follow a systematic and precisely clear protocol in his study. Liou pointed out that 85% of the participants experienced low to moderate levels of pain and discomfort within the first 6–12 h of the injection. A comparison with our results is not straightforward because of the differences in the material used, the methodology as well as the type of tooth movement (en-masse retraction, mesial movement of molars and levelling and alignment versus canine retraction in ours). Our study could be comparable to El-Timamy et al.’s study who adopted the VAS to measure the variables [14]. They found that pain levels were statistically insignificant between the experimental and control sides in their split-mouth study, which does not agree with our results. The difference could result from injecting the intervention side with PRP while the control with calcium chloride, indicating that pain sensation was related to the injection procedure (needle itself) rather than the material. Pain levels were higher in the first, fourth- and seventh-weeks post injection and this is because of the different applied protocol and the frequency of administrations.

Pain levels accompanied by PRP injection have been studied by El Gazzar et al. [28], at different time points 1 h, 6 h, 12 h and 24 h following upper canines’ retraction in a split-mouth design study, they revealed that the values were higher in the study group compared to controls during all the assessed points. However, no pain was detected bilaterally after 24 h which is in accordance with our research. The submucosal tunnel technique injection of PRP has been adopted for the purpose of en-masse retraction in Chandak and Patil’s study [29] who demonstrated increased 24-h-pain levels in the intervention group versus control, unlike after 7 days which showed insignificant difference between both groups, the elevated pain values after 24 h (that contradicted our results) could be explained by the method used for PRP administration which can be considered painful.

In our study, the intensity of pain and discomfort observed at T1 were similar to values detected by Kuroda et al. who registered the highest level of pain and discomfort after one hour of the mini-screw placement [50]. Despite that females were reported to have different pain profiles when compared to males [51] no statistically significant correlations were found between age/gender and their effect on pain/discomfort level in our study.

Swelling levels were greater on the experimental side within the first six hours (*P*-value ≤ 0.01) than the control, then decreased sharply after 24 h and this might be attributed to the oedema associated with the injection and the submucosal accumulation of the material that diminished gradually. This is consistent with Liou’s study [49] who showed that 85% of patients suffered from oedema during 6 to 12 h post injection. Sreenivasagan et al. have evaluated in their study the associated discomfort with different sites of mini-screw insertion and their detrimental effects on chewing and all jaw functions using Wong-Baker Faces Pain rating scale [51], and they found out that difficulty in eating was experienced the most at the infra-zygomatic crest mini-screw region followed by the palatal ones and buccal shelf mini-implants. The interradicular mini-implants had the least score regarding chewing difficulties and the all assessed variables, meaning that they are characterized by the highest acceptability as well as the minimum interferences with daily functions and above all they are preferable over other mini-screw types. Likewise, statistically significant mastication difficulties were detected in our investigation and this could be the result of the accompanying pain resulting from the injection in addition to the discomfort caused by the springs besides food stickiness between the helices of the coil spring that is critical to be cleaned [45], the later reduction and negligible differences were due to the decreased associated levels of pain and discomfort and because patients got used to the coils.

Our findings regarding swallowing and jaw movement limitations demonstrated that there were no statistically significant increases at all time intervals (*P*-value > 0.05). However, the slightly elevated values during the first two hours were explained by the correlated pain and discomfort that quickly faded away, and to the conservative nature of the process unlike the cortical bone puncturing techniques that cause serious amounts of annoyance and require some time to be healed when compared to the injection only.

Satisfaction rates were recorded after the second injection as the patient had a complete perception of the injection procedures and was accustomed to the presence of the coil springs. Fairly good incidence of satisfaction has been reported (75.7%) amongst participants, and this can be attributed to the little-invasive approach which resembles the infiltrative nature of the anaesthesia and is of limited aggressiveness. Eighty-one percent of the patients admitted that extraction of the premolars was more annoying than the injection itself. Our findings were in line with Ganzer et al.’s study who stated that patients’ pain perception after premolar extraction was more negative when compared to the application of miniscrews [52]. Patients were asked to report whether or not they recommend this procedure to a friend and 85.7% of the answers were in favour of recommendation which reflects the good acceptance of this technique and its limited short-term disturbances.

The split-mouth design and not having placebo injections might be considered as the main limitations of this study that could somehow confound our findings. Still, that placebo option would have been unethical and would have caused possible unjustifiable pain from a therapeutic point of view, had it been applied, and hence it was abandoned. Long-term effects of the injection on patients’ perceptions could be addressed when having prolonged follow-up periods, however, all values dropped down to zero at the fifth evaluation time point. Apparently, blinding was only ensured when analysing the results because it was not possible to be employed whether to the main investigator who conducted the research or to the patients who received the injections.

## Conclusions

The results of this study support the following findings:

- Injectable platelet-rich fibrin (i-PRF) doesn’t cause high levels of discomfort within the first day following the injection and it can be considered as a non-invasive technique that causes minimum side effects.
- Platelet-rich fibrin injections are accompanied initially by low to medium pain, discomfort, swelling, eating and swallowing difficulties, jaw movement restriction only 6 h post application.
- Patients treated with this method can get back to their normal life on the second day and the associated values of pain and discomfort drop down to zero by that time meaning that the unfavourable effects of the injection are temporary.

## Data Availability

The datasets used and/or analysed in this study are available from the corresponding author on reasonable request.

## Abbreviations

CONSORT: Consolidated Standards of Reporting Trials
IRB: Institutional Review Board
NSAID: Non-Steroidal Anti-Inflammatory Drugs
OTM: Orthodontic Tooth Movement
IQR: Interquartile Range (Q1-Q3)
i-PRF: Injectable Platelet-Rich Fibrin
Max: Maximum
Min: Minimum
mL: Milli Litre
mm: Millimetre
PROMs: Patient-Reported Outcome Measures
PRP: Platelet-Rich Plasma
QoL: Quality of Life
rpm: Revolutions per Minute
SD: Standard Deviation
VAS: Visual Analogue Scale
